# Spiritual Rejuvenation: An Alternative Way to Healthy Aging

**DOI:** 10.14336/AD.2024.1375

**Published:** 2025-11-02

**Authors:** Dalmacito A. Cordero

**Affiliations:** Department of Theology and Religious Education, De La Salle University, 1004 Manila, Philippines

**Dear Editor,**

I read with great interest a recent article published in this journal regarding the wide-ranging benefits of physical exercise or activity to human health, including improved mental health and the prevention of lifestyle-related diseases. The authors summarized and discussed the potential of physical exercise on counteracting aging-associated white matter demyelination, which causes cognitive decline in Alzheimer’s disease (AD) [1]. I support this claim and echo how physical activity can affect positively in many ways one’s mental health and promote health aging for older adults. On the other hand, I argue that physical activity may not be applicable to all cases due to various reasons related to physical limitations. Thus, I propose an alternative intervention which are focused on spiritual rejuvenation (SR).

Physical activity (PA) is often seen as an intervention that has the potential of decreasing the burden associated with depression and cognitive impairment in later life [2]. Some older adults often are more likely to engage in PA in lower intensity, such as walking, stretching, gardening, riding a bicycle, playing golf, and other sports. Others engaged in nature-based intervention or travel therapy which can positively impacts feelings, emotions, and mood. These nature sessions produced restorative experiences that mediated the decrease in depression in addition to standard care [3]. However, some older people with mobility problems may find this difficult and not doable. As an alternative, I propose some practices that aims to enhance the spirituality of older adults through spiritual rejuvenation (SR) practices. SR practices may require little physical effort, and it is anchored on one’s expression of spirituality. Spirituality is a subjective, intangible, and multifaceted aspect in human beings. It is sometimes associated with religion and is used interchangeably. Spirituality involves one’s discovery of a meaningful life, while religion involves structures, rituals, and practices that lead a person to a higher power or God [4]. Though these concepts are not strictly similar, the practices under each realm are meant to uplift the spiritual aspect of older people and their wellbeing. According to Dr. Gala Gorman, a holistic life coach and author of *Spiritual Approach*, performing spiritual activities is considered a self-care practice that helps people improve their mental and emotional health. These practices may include prayer, contemplation, spending time in nature, non-judgement, regular acts of compassion, silence, letting go, chanting, meditation, and many others. Another study reported that positive spirituality plays a vital role in dealing with the sick persons’ feeling of helplessness and loss of control. Spiritual activities, like prayer, can help adults to reduce feelings of isolation, especially the presence of a community surrounding them [5]. Lastly, the belief or faith in God, as grounded in one's religion, offers OP on being secured and hopeful after their earthly life, letting them prepare for their future deaths with acceptance and less fear [6]. Aside from these SR practices, other simple social interactions and enjoying hobbies, such as book reading, handcrafting and knitting, can keep one's mental status healthy, and thus contribute to maintaining one's cognitive functions as PAs do. I hope that more scientists and researchers will be interested and engaged in studying on the relationships between SR and healthy brain aging.

Health is a fundamental human right. Regardless of age, everyone should be entitled to control one's health and body for healthy living. Healthy aging must not be taken away from older people. The love and care that they have given us must also be returned to them in multitude. We will all age, but we must age with grace and in good health, having lived life to the full.
